# Redução na Biodisponibilidade Sistêmica de Óxido Nítrico Concomitante à Disfunção Endotelial Microvascular durante o Bypass Cardiopulmonar

**DOI:** 10.36660/abc.20201040

**Published:** 2021-09-01

**Authors:** Viviana Ugenti, Ana Catarina Romano, Andrea De Lorenzo, Eduardo Tibirica

**Affiliations:** 1 Instituto Nacional de Cardiologia Rio de Janeiro RJ Brasil Instituto Nacional de Cardiologia,^1^ Rio de Janeiro, RJ - Brasil

**Keywords:** Fluxometria por Laser-Doppler, Óxido Nítrico, Ponte Cardiopulmonar

## Introdução

O bypass cardiopulmonar (BCP) é atualmente realizado em lactentes e recém-nascidos para correção cirúrgica de cardiopatias congênitas (CCs).^1^ O procedimento expõe o corpo a condições não fisiológicas extremas, que iniciam uma resposta inflamatória sistêmica acompanhada por disfunção vasomotora, e podem levar à disfunção múltipla de órgãos.^2^ Além disso, o BCP está relacionado à ativação e lesão de células endoteliais, que se está associada à resposta inflamatória global, desencadeamento do sistema de coagulação e subsequente disfunção de órgãos, não somente em pacientes adultos, mas particularmente em lactentes e recém-nascidos.^3^

A disfunção microvascular sistêmica durante o BCP resulta em fluxo sanguíneo, perfusão de órgãos, e oxigenação tecidual inadequados.^4^ Nós mostramos, utilizando monitoramento de perfusão por laser Doppler (LDPM, do inglês laser Doppler perfusion monitoring), que consiste em um método não invasivo acoplado com aquecimento local da pele, que o leito microcirculatório da pele da testa é um modelo apropriado para o estudo da reatividade microvascular e perfusão tecidual na cirurgia cardiovascular com BCP em adultos.^5^ De fato, a hiperemia térmica local (HTL) é um método útil na avaliação da função endotelial microvascular sistêmica.^5^^,^^6^ Além disso, utilizando LDPM, demonstramos a ocorrência de disfunção microvascular e hipoperfusão durante o BCP na correção cirúrgica de doença cardíaca congênita em lactentes e crianças, apesar de parâmetros macrohemodinâmicos adequados.^7^ O descompasso entre a microcirculação e a macrocirculação em pacientes graves com choque séptico ou cardiogênico, o que pode prejudicar o manejo clínico adequado desses pacientes, tem estimulado a busca por novos métodos para monitorar a perfusão da microcirculação em unidades de terapia intensiva.^8^

Vale ressaltar que a liberação de óxido nítrico (NO) a partir do óxido nítrico-sintase endotelial (eNOS) está reduzida durante o BCP em pacientes adultos.^9^ O NO é um potente vasodilatador derivado do endotélio, e sua depleção durante BCP sob fluxo não pulsátil pode levar à vasoconstrição, e consequentemente à diminuição na perfusão de órgãos. NO_x_ (NO_2_^–^ / NO_3_^–^) plasmáticos, metabólitos estáveis do NO, têm sido usados como marcadores da biodisponibilidade de NO sistêmico, uma vez que seus níveis refletem mudanças na atividade de eNOS em humanos.^10^ Assim, o objetivo do presente estudo foi investigar se uma redução na biodisponibilidade sistêmica de NO está associada com disfunção microvascular dependente do endotélio em lactentes e crianças, durante cirurgia cardíaca com circulação extracorpórea para correção de CC acianótica.

## Métodos

Este estudo observacional longitudinal incluiu 47 pacientes pediátricos consecutivos com CCs acianóticas, com idade entre um mês e nove anos, submetidos à cirurgia cardíaca corretiva em um hospital público terciário no Brasil. O estudo foi conduzido de acordo com a declaração de Helsinki, e foi aprovado pelo comitê de ética da instituição. Os pais ou os responsáveis legais pelos participantes do estudo assinaram um termo de consentimento. Os procedimentos anestésicos ocorreram sob BCP, com hipotermia leve a moderada (32-34° C). A pressão arterial média foi mantida entre 45 e 60mmHg.

### Avaliação do fluxo e reatividade microvascular

A reatividade microvascular da pele foi avaliada usando um sistema de LDPM (Periflux 5001, Perimed, Järfälla, Suécia), que mede, em um único ponto, o fluxo microvascular utilizando uma sonda laser para aquecimento (PF 457, Perimed). Mudanças na perfusão microvascular foram registradas em Unidades Arbitrárias (UA = 10mV). A sonda foi posicionada na testa no início dos procedimentos anestésicos e o fluxo microvascular basal medido durante 20 minutos de aquecimento local da sonda laser a 42oC (HTL). A vasodilatação máxima foi expressa em Condutância Vascular Cutânea, calculada como a razão entre fluxo microvascular, em UA, e a pressão arterial média (UA/mmHg). Os valores médios de fluxo microvascular (em UA) antes e durante o BCP foram usados nos cálculos. Após a avaliação basal, a resposta microvascular à HTL também foi registrada após indução da anestesia geral e 15 minutos após início do BCP.

### Avaliação da biodisponibilidade sistêmica do NO

A biodisponibilidade sistêmica do NO foi avaliada utilizando concentrações plasmáticas de NOx (NO2- / NO3-), que foram usadas como um índice de formação de NO in vivo. As concentrações plasmáticas de NOx foram determinadas após indução anestésica e imediatamente após conclusão de BCP usando um teste colorimétrico (Cayman Chemical Company, Ann Arbor, Michigan, EUA) com sensibilidade de 2,5μM, e um coeficiente de variação intraensaio de 2,7%. Cada amostra de plasma foi medida em duplicata.

### Análise estatística

Os resultados foram apresentados como medianas (intervalo interquartil). O teste de Shapiro-Wilk foi usado para testar a normalidade dos dados. Os resultados foram analisados com teste dos postos sinalizados de Wilcoxon bicaudal para amostras pareadas, usando o programa GraphPad Prism 7.0 (GraphPad Software INC., San Diego, California, EUA). Um valor de p < 0,05 foi considerado estatisticamente significativo.

## Resultados

**Figura 1:**
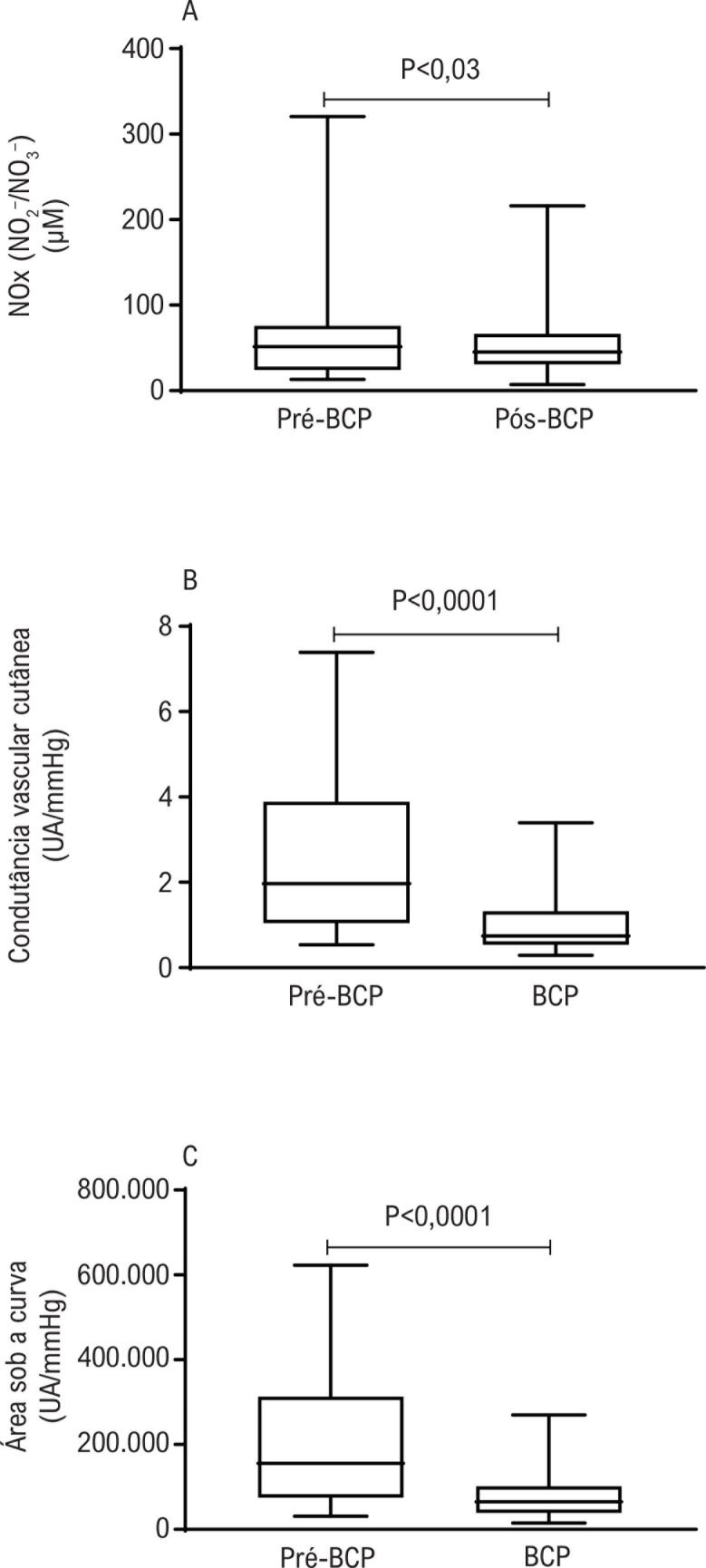
(A) Concentrações plasmáticas totais de NO_x_ (NO_2_^–^ / NO_3_^–^) antes do bypass cardiopulmonar (BCP) (pré-BCP) e após o BCP (pós-BCP). (B) picos de resposta de vasodilatação microvascular da pele induzida por hiperemia térmica local, expressa por condutância vascular cutânea [unidades arbitrárias (UA)/pressão arterial média (mmHg)] antes do BCP (pré-BCP) e 15 minutos após o início do BCP. (C) Área sob a curva das respostas vasodilatadoras induzidas por hiperemia térmica local antes do BCP (pré-BCP) e 15 minutos após o início do BCP. Valores expressos em diagrama de caixa e limites inferior e superior, em que a linha central representa o valor mediano, a caixa contém os percentis 25 e 75 do conjunto de dados. Resultados analisados pelo teste dos postos sinalizados de Wilcoxon para amostras pareadas.

As características basais dos pacientes incluídos no estudo e os parâmetros cirúrgicos estão apresentados na Tabela 1. Os valores médios de pressão arterial foram 58,4 ± 11,5 mmHg antes do BCP e 50,9 ± 8,4 mmHg durante o BCP (p=0,01). Antes do BCP, os níveis plasmáticos de NOx foram 51,4 (24,2-75,8) µM, os quais foram reduzidos significativamente para 45,1 (31,0-66,5) µM após BCP (p=0,03; Figura 1A).

**Tabela 1:** Características clínicas e dados cirúrgicos dos pacientes (n=47)

Parâmetros		
Sexo masculino n (%)		19 (40,4)
Idade (meses)		16 (9 - 54)
Peso (Kg)		8 (6 - 16)
Tempo de BCP (min)		85 (75 - 105)
Temperatura durante o BCP (°C)		32 (32-33)
Taxa de fluxo da bomba (mL/Kg/min)		150 (120 - 150)
Tempo de clampeamento aórtico (min)		67 ± 28
**Tipo de cardiopatia n (%)**		
	Defeito do septo atrial	3 (6,4)
Defeito do septo ventricular		16 (34)
	Canal atrioventricular (parcial ou total)	15 (32)
	Interrupção do arco aórtico	1 (2,1)
	Truncus arteriosus	1 (2,1)
Lesões mistas		11 (23,4)
**Escore RACHS-1 n (%)**		
Categoria de risco 1		3 (6,4)
Categoria de risco 2		21 (44,7)
Categoria de risco 3		21 (44,7)
Categoria de risco 4		2 (4,2)

Dados apresentados em média ± desvio padrão ou medianas (percentis 25 - 75) para valores que não apresentaram distribuição gaussiana (teste de normalidade de Shapiro-Wilk); BCP: bypass cardiopulmonar; RACHS-1: escore de risco ajustado para cirurgia de cardiopatia congênita

Não houve mudança significativa na condutância microvascular basal mediana antes do BCP [0,47 (0,35-0,64) APU/mmHg] e durante do BCP (p=0,85). Por outro lado, o aumento dependente do endotélio na condutância microvascular, induzido pela hiperemia térmica, foi reduzido durante o BCP em comparação aos valores obtidos antes do BCP (Figura 1B). Os valores absolutos máximos da condutância microvascular durante a HTL antes do BCP [1,97 (1,04-3,89) UA/mmHg] foram significativamente reduzidos para [0,74 (0,54-1,32) UA/mmHg] durante o CBP (p<0,0001; Figura 1B). Ainda, observou-se uma importante redução nos aumentos na porcentagem de condutância microvascular induzidos por HTL, de [313 (171-604) %] antes do BCP para [74 (8-156) %] durante o BCP (p<0,0001). A área sob a curva (AUC) da vasodilatação microvascular induzida por HTL mostrou um padrão similar de resposta. Valores máximos de aumento na AUC induzido por HTL antes do BCP [155 552 (75 323-313 040) UA/mmHg/s] foram significativamente reduzidos para [64 676 (38 753-101 423) UA/mmHg/s] durante o CBP (p<0,0001; Figura 1C).

## Discussão

O NO tem um papel chave na regulação da função endotelial e inflamação microvascular.11 O BCP induz uma resposta inflamatória generalizada, induzida, ao menos em parte, pela lesão de isquemia/reperfusão, a qual contribui para disfunção miocárdica e débito cardíaco reduzido, e está relacionada ao metabolismo do NO, entre outros mecanismos.^12^ O presente estudo mostra uma redução nos níveis de NO durante o BCP em crianças, concomitante à evidente disfunção de disfunção microvascular. Tal fato destaca evidências prévias de redução de NO durante cirurgia cardíaca,^13^ o que tem motivado o desenvolvimento de estudos usando administração de NO para reduzir a inflamação induzida por bypass em crianças submetidas à cirurgia cardíaca.^14^ Portanto, dados deste estudo possivelmente corroboram o monitoramento da microcirculação durante o BCP, com intervenções terapêuticas direcionadas.

## Conclusões

A disfunção endotelial microvascular durante o BCP na cirurgia cardíaca para a correção de CCs parece estar relacionada a uma biodisponibilidade sistêmica reduzida de NO, resultante de uma resposta inflamatória e pró-oxidativa típica desse procedimento cirúrgico.

Importante mencionar que nosso estudo teve um delineamento experimental transversal e, portanto, a reatividade da microcirculação na nossa população foi avaliada sem nenhuma intervenção. No entanto, considerando que nossos resultados apontaram para a existência de uma associação entre BCP e depleção sistêmica de NO em lactentes e crianças, pretendemos estender nosso estudo clínico com intervenções medicamentosas – tais como nitroprussiato de sódio, uma droga doadora de NO – usadas para otimizar a microcirculação durante o BCP, para investigar seus supostos efeitos benéficos sobre as mudanças na microcirculação. Por hora, o LDPM refletiu mudanças na perfusão e na reatividade microvascular que se correlacionaram bem com alterações nos perfis do fluxo, pressão de perfusão e disfunção endotelial, induzidos por uma síndrome de resposta inflamatória sistêmica.

Por fim, nós sugerimos que o uso de monitoramento da microcirculação durante a cirurgia cardíaca, com implementação de variáveis relacionadas à microcirculação/perfusão tecidual na prática de rotina durante o BCP, junto com intervenção terapêutica adequada na microcirculação, tem o potencial de melhorar desfechos na cirurgia cardíaca pediátrica.
