# Supplementary material for: Redução na Biodisponibilidade Sistêmica de Óxido Nítrico Concomitante à Disfunção Endotelial Microvascular durante o Bypass Cardiopulmonar
Source: Arq Bras Cardiol. 2021 Sep 1;117(3):554–7. [Article in Portuguese] doi: 10.36660/abc.20201040 (PMC8462954; doi:10.36660/abc.20201040)
Supplement: Translation

# Translation: Reduced Systemic Nitric Oxide Bioavailability Parallels Microvascular Endothelial Dysfunction during Cardiopulmonary Bypass

**Authors:** Viviana Ugenti, Ana Catarina Romano, Andrea De Lorenzo, Eduardo Tibirica

**Affiliations:** 1 Rio de Janeiro RJ Brazil Instituto Nacional de Cardiologia,^1^ Rio de Janeiro, RJ - Brazil

**Keywords:** Laser-Doppler Flowmetry, Nitric Oxide, Cardiopulmonary Bypass

## Introduction

Cardiopulmonary bypass (CPB) is currently performed in infants and newborns for surgical correction of congenital heart diseases (CHDs).^1^ CPB exposes the body to extreme, nonphysiologic conditions that initiate a systemic inflammatory response accompanied by vasomotor dysfunction, and can lead to multiple organ dysfunction.^2^ Additionally, CPB has been linked to activation and injury of endothelial cells, which is associated with global inflammatory response, triggering of the coagulation system and subsequent organ dysfunction, not only in adult patients, but particularly in infants and newborns.^3^

Systemic microvascular dysfunction during CPB results in inadequate blood flow, organ perfusion and oxygen delivery to the tissues.^4^ We have shown, using laser Doppler perfusion monitoring (LDPM) (a non-invasive method coupled with local heating of the skin), that the microcirculatory bed of the skin of the forehead is an appropriate model for the study of microvascular reactivity and tissue perfusion in cardiovascular surgery with CPB in adults.^5^ Actually, local thermal hyperemia (LTH) is a useful method in the evaluation of systemic microvascular endothelial function.^5^^,^^6^ In addition, using LDPM, we demonstrated that microvascular dysfunction and hypoperfusion occur during CPB in surgical correction of congenital heart disease in infants and children, despite adequate macrohemodynamic parameters.^7^ The mismatch between macro- and microcirculation in severely ill patients with septic or cardiogenic shock, which can be misleading to an adequate clinical management of these patients, has also stimulated the search for new methods to monitor microcirculatory perfusion in the intensive care units.^8^

Of note, the release of nitric oxide (NO) from the constitutive endothelial isoform of nitric oxide synthase (eNOS) is reduced during CPB in adult patients.^9^ NO is a potent endothelium-derived endogenous vasodilator, and its depletion during CPB under non-pulsatile flow can lead to vasoconstriction, and consequently reduced organ perfusion. Plasma NO_x_ (NO_2_^–^ / NO_3_^–^), steady metabolites of NO, which have been used as markers of systemic NO bioavailability, as their levels reflect changes in eNOS activity in humans.^10^ Therefore, the aim of the present study was to investigate whether a reduction of NO systemic bioavailability is associated with endothelial-dependent microvascular dysfunction in infants and children, during on-pump cardiac surgery for the correction of acyanotic CHD.

## Methods

This longitudinal observational study included 47 consecutive pediatric patients with acyanotic heart defects, aged between 1 month and 9 years, undergoing corrective cardiac surgery at a tertiary public Brazilian hospital. The study was undertaken according to the Declaration of Helsinki and was approved by the ethics committee of the institution. Parents or legal tutors of the study participants gave written informed consent. The anesthetic procedures occurred under CPB, with mild to moderate hypothermia (32-34° C). Mean arterial pressure was kept between 45-60 mmHg.

### Assessment of microvascular flow and reactivity

Skin microvascular reactivity was evaluated using a single-point LDPM system (Periflux 5001, Perimed, Järfälla, Sweden), which measures microvascular flow using a heating laser probe (PF 457, Perimed). The microvascular perfusion changes were registered in arbitrary perfusion units (APU=10 mV). The probe was positioned on the forehead at the beginning of the anesthetic procedures, and baseline microvascular flow was measured during 20 minutes of local heating of the laser probe to 42 °C (LTH). The maximal vasodilation was expressed as cutaneous vascular conductance (CVC), calculated as the ratio of the microvascular flow, in APU, to the mean arterial pressure (APU/mmHg). Mean of values of microvascular flow (in APU) before and during CPB were used in the calculations. After the baseline evaluation, the microvascular response to LTH was also recorded after induction of general anesthesia and 15 minutes after the beginning of CPB.

### Evaluation of systemic bioavailability of NO

Systemic nitric oxide bioavailability was evaluated using plasma NOx (NO_2_^–^ / NO_3_^–^) concentrations, which have been used as an index of in vivo NO formation. Total plasma NOx concentrations were determined after induction of anesthesia and immediately after conclusion of CPB using a colorimetric assay (Cayman Chemical Company, Ann Arbor, Michigan, USA) with a 2.5 μM sensitivity and a 2.7% intra-assay coefficient of variation. Each plasma sample was measured in duplicate.

### Statistical analysis

Results are presented as medians (interquartile range). Shapiro-Wilk was used to test the normality of the data. Results were analyzed with two-tailed Wilcoxon matched-pairs signed rank test using GraphPad Prism 7.0 software (GraphPad Software INC., San Diego, California, USA). A p-value < 0.05 defined statistical significance.

## Results

Baseline clinical characteristics of the patients included in the study and the surgical parameters are depicted in Table 1. Mean arterial pressure values were 58.4 ± 11.5 mmHg before and 50.9 ± 8.4 mmHg during CPB (p=0.01).

**Table 1:** Clinical characteristics and surgical data of patients (n=47)

Parameters		
Male gender n (%)		19 (40.4)
Age (months)		16 (9 - 54)
Weight (Kg)		8 (6 - 16)
CPB time (min)		85 (75 - 105)
Temperature during CPB (°C)		32 (32-33)
Pump flow rate (mL/Kg/min)		150 (120 - 150)
Aortic cross-clamping time (min)		67 ± 28
**Type of cardiopathy n (%)**		
	Atrial septal defect	3 (6.4)
Ventricular septal defect		16 (34)
	Atrioventricular canal (partial or total)	15 (32)
	Aortic arch interruption	1 (2.1)
	Truncus arteriosus	1 (2.1)
Mixed lesions		11 (23.4)
**RACHS-1 score n (%)**		
Risk category 1		3 (6.4)
Risk category 2		21 (44.7)
Risk category 3		21 (44.7)
Risk category 4		2 (4.2)

Dados apresentados em média ± desvio padrão ou medianas (percentis 25 - 75) para valores que não apresentaram distribuição gaussiana (teste de normalidade de Shapiro-Wilk); BCP: bypass cardiopulmonar; RACHS-1: escore de risco ajustado para cirurgia de cardiopatia congênita

Before CPB, patients' plasma NOx levels were 51.4 (24.2-75.8) µM, which were significantly reduced to 45.1 (31.0-66.5) µM after CPB (p=0.03; Figure 1A).

**Figure 1:** (A) Total plasma levels of NO_x_ (NO_2_^–^ / NO_3_^–^) before (PRE-CPB) and after the termination of cardiopulmonary bypass (POST-CPB). (B) Peak values of skin microvascular vasodilator responses induced by local thermal hyperemia, expressed as cutaneous vascular conductance [arbitrary perfusion units (APU)/mean arterial pressure (mmHg)] before (PRE-CPB) and 15 minutes after the beginning of CPB. (C) Area under the curve of vasodilator responses induced by local thermal hyperemia before (PRE-CPB) and 15 minutes after the beginning of CPB. Values are expressed as box and whisker plots where the center line denotes the median value, the box contains the 25^th^ to 75^th^ percentiles of dataset and whiskers mark the maximum and minimum values. Results were analyzed using the Wilcoxon matched-pairs signed rank test

Median baseline microvascular conductance before CPB [0.47 (0.35-0.64) APU/mmHg] did not change significantly [0.48 (0.28-0.66) APU/mmHg] during CPB (p=0.85). On the other hand, the endothelial-dependent increase in microvascular conductance, induced by thermal hyperemia, was significantly blunted during CPB, when compared to values obtained before CPB (Figure 1B). Maximum absolute values of microvascular conductance during LTH before CPB [1.97 (1.04-3.89) APU/mmHg], were markedly reduced to [0.74 (0.54-1.32) APU/mmHg] during CPB (p<0.0001; Figure 1B). Percentage increases of microvascular conductance induced by LTH were also markedly reduced, from [313 (171-604) %] before CPB to [74 (8-156) %] during CPB (p<0.0001). The area under the curve (AUC) of microvascular vasodilation induced by LTH showed a similar pattern of response. Maximum increases in AUC induced by LTH before CPB [155,552 (75,323-313,040) APU/mmHg/s] were significantly reduced to [64,676 (38,753-101,423) APU/mmHg/s] during CPB (p<0.0001; Figure 1C).

## Discussion

NO has a key role in the regulation of endothelial function and microvascular inflammation.^11^ CPB induces a generalized inflammatory response, triggered at least in part by ischemia–reperfusion injury, which contributes to myocardial dysfunction and reduced cardiac output, and is related to NO metabolism, among other mechanisms.^12^ The current study shows a reduction of NO during CPB in children, which parallels the objective evidence of microvascular dysfunction. This underscores prior evidence of NO reduction during cardiac surgery,^13^ what has led to studies using NO administration to reduce bypass-induced inflammation in children undergoing cardiac surgery.^14^ Therefore, data from this study may support the use of microcirculatory monitoring during CPB, with guided therapeutic interventions.

## Conclusions

The impairment of microvascular endothelial function during CPB in cardiac surgery for the correction of congenital heart defects appears to be related to a reduced systemic bioavailability of NO, resulting from the inflammatory and pro-oxidative response typical of this surgical procedure.

It is important to note that our study had a cross-sectional experimental design; thus, microcirculatory reactivity in our population was evaluated without any intervention. Nevertheless, considering that our results pointed out to the existence of an association between CPB and systemic NO depletion in infants and children, we intend to extend our clinical research with drug interventions - such as sodium nitroprusside, a NO donor drug - utilized for optimizing microcirculation during CPB, to investigate their putative beneficial effects on microcirculatory alterations. For the moment, LDPM reflected changes in microvascular perfusion and reactivity that correlated well with shifts in flow patterns, perfusion pressure and endothelial dysfunction, triggered by the systemic inflammatory response syndrome.

Finally, we suggest that the use of microcirculatory monitoring during cardiac surgery, with the implementation of microcirculatory/tissue perfusion variables in routine care during CPB, together with appropriate therapeutic microcirculatory intervention, has the potential to improve outcomes in pediatric cardiac surgery.
